# Beta-Hydroxybutyrate: A Dual Function Molecular and Immunological Barrier Function Regulator

**DOI:** 10.3389/fimmu.2022.805881

**Published:** 2022-06-16

**Authors:** Jiancheng Qi, Linli Gan, Jing Fang, Jizong Zhang, Xin Yu, Hongrui Guo, Dongjie Cai, Hengmin Cui, Liping Gou, Junliang Deng, Zhisheng Wang, Zhicai Zuo

**Affiliations:** ^1^ Key Laboratory of Animal Disease and Human Health of Sichuan Province, College of Veterinary Medicine, Sichuan Agricultural University, Chengdu, China; ^2^ Institute of Animal Nutrition, Sichuan Agricultural University, Chengdu, China

**Keywords:** ketone bodies, beta-hydroxybutyrate, endogenous ketogenesis, dual-function molecular, immunoregulation, immunological barrier, mucosa, innate immune system

## Abstract

Ketone bodies are crucial intermediate metabolites widely associated with treating metabolic diseases. Accumulating evidence suggests that ketone bodies may act as immunoregulators in humans and animals to attenuate pathological inflammation through multiple strategies. Although the clues are scattered and untrimmed, the elevation of these ketone bodies in the circulation system and tissues induced by ketogenic diets was reported to affect the immunological barriers, an important part of innate immunity. Therefore, beta-hydroxybutyrate, a key ketone body, might also play a vital role in regulating the barrier immune systems. In this review, we retrospected the endogenous ketogenesis in animals and the dual roles of ketone bodies as energy carriers and signal molecules focusing on beta-hydroxybutyrate. In addition, the research regarding the effects of beta-hydroxybutyrate on the function of the immunological barrier, mainly on the microbiota, chemical, and physical barriers of the mucosa, were outlined and discussed. As an inducible endogenous metabolic small molecule, beta-hydroxybutyrate deserves delicate investigations focusing on its immunometabolic efficacy. Comprehending the connection between ketone bodies and the barrier immunological function and its underlining mechanisms may help exploit individualised approaches to treat various mucosa or skin-related diseases.

## Introduction

**Figure 1:**
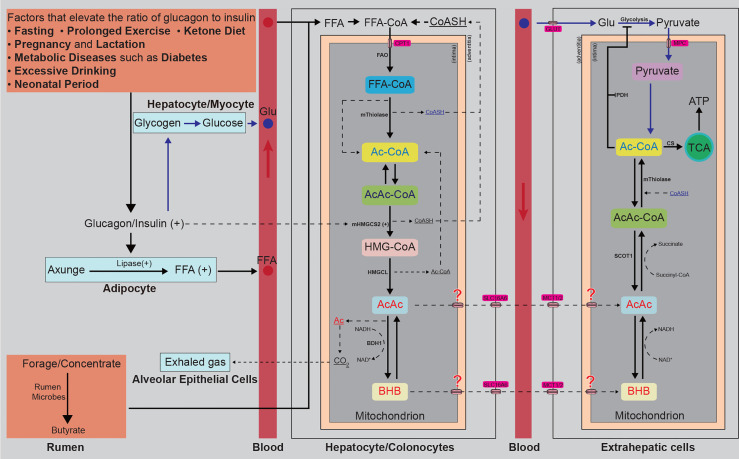
Diagrammatic sketch of endogenous generation and consumption of BHB. Any factors that elevate the ratio of glucagon to insulin, such as fasting, prolonged exercise, insisting on a ketone diet, lactation, diabetes, or alcoholism, can accelerate the process of steatolysis and release lots of FFAs. The released FFAs are transported to the liver by albumin and are condensed with free CoASH in hepatocyte plasma. Then the condensed FFAs are transported into the mitochondrion by CPT1. The forage and concentrate englobed by ruminants are fermented into butyrate by rumen microbes and absorbed by colonocytes. The condensed FFAs and butyrate transported into the mitochondrion of hepatocytes or colonocytes are β-oxidated into Ac-CoA, two of which are further condensed into an AcAc-CoA, releasing a CoASH. The Ac-CoA and AcAc-CoA are then catalyzed to condense into HMG-CoA by mHMGCS2, encoded by a strictly spatiotemporally controlled gene (*mHMGCS2*). The HMG-CoA is cleaved into Ac-CoA and AcAc by HMGCL. The AcAc is reduced into D-BHB by BDH1 (consuming NADH and releasing NAD^+^) or spontaneously decarboxylated to volatile acetone and CO^2^, released into the bloodstream by free diffusion and easily eliminated through the alveolar epithelial cells in the lung. Ac and AcAc are released into circulating blood. The D-BHB is transported across the mitochondrial intima through a yet-unknown mechanism and across the mitochondrial adventitia by SLC16A6 and released into the circulating blood. AcAc and BHB are absorbed by extrahepatic cells. BHB is oxidated to AcAc by BDH1 (consuming NAD^+^ and releasing NADH, a reversible reaction that also occurs in the generation process of BHB), and AcAc is activated into AcAc-CoA by SCOT1 (succinyl-CoA donates the CoA), and then split into Ac-CoA by mThiolase, consuming CoASH. The glucose derived from the glycogen is transported to and absorbed into the extrahepatic cells by GLUT. In the cytoplasm, the glucose is oxidated into pyruvate by glycolysis, inhibited by the BHB-derived Ac-CoA. Pyruvate is transported into the mitochondrion and then dehydrogenated to Ac-CoA by PDH, inhibited by the BHB-derived Ac-CoA. The BHB and pyruvate-derived Ac-CoA enter the TCA circle or act as signalling molecules. CoASH, coenzyme A; FFAs, free fatty acids; CPT1, carnitine palmitoyltransferase 1; FAO, fatty acids β-oxidation; Ac-CoA, acetyl-CoA; AcAc-CoA, acetoacetyl-CoA; mHMGCS2, mitochondrial 3-hydroxymethylglutaryl-coenzyme A synthase; HMG-CoA, hydroxymethylglutaryl-CoA; HMGCL, hydroxymethylglutaryl coenzyme A lyase; Ac, acetone; AcAc, acetoacetate; BHB, β-hydroxybutyrate; NADH/NAD^+^, nicotinamide adenine dinucleotide; SLC16A6, solute carrier family 16 member 6; MCT, monocarboxylate transporters; OXCT1, 3-oxoacid-CoA transferase 1; GLU, glucose; GLUT, glucose transporter; MPC, membrane permeation channel; CS, Citrate Synthase; TCA, tricarboxylic acid; ATP, adenosine triphosphate.

As a kind of indispensable spare metabolic fuel source, ketone bodies, including beta-hydroxybutyrate (BHB), acetone (Ac), and acetoacetate (AcAc), are accepted to play important roles in all realms of life and attract much attention by many researchers (1–5). Factors such as fasting, prolonged exercise, and feeding a ketogenic diet will increase the endogenous hepatic ketogenesis, which significantly raises the total concentration of ketone bodies in circulating blood and tissues. These ketone bodies are thought to participate in the overall energy metabolism within extrahepatic tissues and replenish energy (6) (**Figure 1**). For instance, humans and cows frequently undergo energy deficiency situations in which they utilize ketone bodies as the major energy fuel. In healthy humans, the concentration of total circulating ketone bodies exhibits circadian oscillations (0.1~0.2 mM), raises to approximately 1mM after 24 hours of fasting or prolonged exercise, and even up to 20 mM in pathological states like diabetic ketoacidosis (7). Since ketone bodies possess high permeability to the blood-brain barrier (BBB), especially during a low glucose accession period, they become the principal energy source for the brain because the glucose supplement is inadequate (8, 9). Dairy cows often suffer from serious starvation because of the massive metabolic demands to support their lactation needs. It was reported that approximately 45% of dairy cows had circulating BHB above thresholds associated with metabolic diseases, such as bovine subclinical ketosis (over 1.7 mM) (10, 11). However, it’s important to realize that elevated circulating BHB has complex physiological consequences and is considered an attractive strategy to treat multiple diseases by some researchers, which is why the ways to elevate circulating ketones exogenously have been developed. For example, oral administration of exogenous BHB supplements, such as D-β-hydroxybutyrate monoester and ketone ester (KE), has become an efficient avenue to elevate circulating BHB concentration (12). Intravenous BHB is also a new approach to boosting the circulation of BHB because it obviates the time delay of circulating BHB elevation induced by oral BHB supplements administration (1, 13).

In recent years, elevated blood ketones concentration by endogenous and exogenous interventions has been widely studied in experimental and clinical research, showing great application potential, including applications in relieving epilepsy (14), Parkinson’s disease (15, 16), gout flares (17), respiratory tract influenza virus infections (18), and obesity (19, 20). Intriguing, almost all these applications are thought to involve immune systems regulations induced by energy and/or signal information carried by BHB (21). Recently, Simone and colleagues directly investigated the yet-unproven assumption that ketone bodies positively affect human immunity and found that a ketogenic diet markedly enhanced the capacities of CD4^+^, CD8^+^, and regulatory T cells, augmented T_mem_ cells formation, consequently improved overall human T-cell immunity (22) even though further deeply and specific studies are warranted. Besides, previous work also showed that intravenous injection of BHB significantly decreased the diversity of microbiota community of the yak nasopharynx mucosa and increased the abundance of some pathogenic bacteria, implying that ketone bodies might influence the function of mucosal barriers (13), which also needs to be further investigated. Although direct evidence is lacking, mounts of indirect testimonies could be collected to support the putative influence of ketone bodies on the immune system. Hence, we outlined and discussed the previous research regarding the effect of ketone bodies on the immunological barriers in this review.

## Endogenous Ketogenesis

The ketone bodies are predominant lipid-derived metabolites produced by the hepatocytes through fatty acid oxidation (FAO) (23). They are also the key nodes connecting multiple metabolic pathways, including β-oxidation, the tricarboxylic acid (TCA) cycle, gluconeogenesis, and the biosynthesis of lipid and sterols (6, 7, 24). Non-hepatic peripheral tissues cannot directly utilize the free fatty acids (FFAs) derived from adipose tissues because of the lack of a key mitochondrial enzyme 3-hydroxymethylglutaryl-coenzyme A synthase (mHMGCS2), which is why they are transported by albumin to the liver, one of the only two tissues abundantly expressing mHMGCS2 (24, 25). In hepatocyte plasma, FFAs are condensed with free coenzyme A (CoASH) and activated (26). They are transported into the mitochondrial *via* carnitine palmitoyltransferase 1 (CPT1), where they are β-oxidated into Ac-CoA and AcAc-CoA. Then the mHMGCS2 catalyzes fatty acid-β-oxidation-derived Ac-CoA and AcAc-CoA condense into hydroxymethylglutaryl (HMG)-CoA, which is cleaved into Ac-CoA and AcAc later by hydroxymethylglutaryl coenzyme A lyase (HMGCL) (27). Most of the AcAc is reduced to D-β-hydroxybutyrate (D-BHB) by phosphatidylcholine-dependent mitochondrial D-BHB dehydrogenase (BDH1) (28–30). A very small part of AcAc can also be spontaneously decarboxylated to volatile acetone and CO_2_, released into the bloodstream by free diffusion and easily eliminated through the lung, explaining the rotten apple smell in the exhaled air from ketosis patients (31). Hence, BHB is the main ketone body in animal circulating blood (over 70%). AcAc and BHB cross the hepatocyte mitochondrial inner membrane through an unknown-yet mechanism and are then released into circulating blood by transporter solute carrier family 16 member 6 (SLC16A6) (32), a member of the monocarboxylate transporters (MCT) protein family (33). As small polar molecules, ketone bodies are soluble in blood, indicating that they can be transported to extrahepatic tissues through the circulation and then imported into extrahepatic cells by MCT1/2 to wield their energy/signaling carrier roles (34). The diagrammatic sketch of endogenous ketogenesis is shown in **Figure 1**.

Of note, it seems that intestinal epithelial cells also contribute to the local ketone bodies pool of animals (35, 36). In addition to the hepatocyte-dependent ketogenesis, as mentioned above, some intestinal epithelial cells were recently reported to abundantly express mHMGCS2 (37, 38), indicating its potential to produce ketone bodies. Indeed, butyrate from polysaccharides fermentation by some anaerobic bacteria is easily absorbed by colonocytes or transported to the liver for ketogenesis (36, 39). This process plays an important role in the differentiation of colonocytes (40). In ruminants, colonocytes-derived BHB affects the development of the rumen and is believed to be associated with the responsiveness to weaning and infections (10, 41, 42).

Fragmentary extrahepatic ketogenesis has been reported in tumour cells (43), astrocytes of the central nervous system (CNS) (44), renal cells (45), retinal pigment epithelium (46), or other cells with an unusual expression of mHMGCS2 (47, 48), ketogenesis was also observed. However, in the physiological state, no extrahepatic tissue presents a higher steady-state ketone body concentration than in the circulation (49), indicating that the extrahepatic ketogenesis does not influence the circulating ketone body concentration. Collectively, by controlling the substrate accession (mainly controlled by the ratio of insulin to glucagon) and strictly spatiotemporal expression of mHMGCS2 (primarily regulated by the fork-head transcription factor), endogenous ketogenesis is delicately controlled (50, 51). Previous work has reviewed the regulations of ketogenesis in detail (34, 52). For now, two queries are still needed to be studied. The first is how the AcAc and BHB cross the mitochondrial adventitia; the other is the physiological action of extrahepatic ketogenesis in bodies.

## Energy and Signal Roles of BHB

Ketone bodies play extensive physiological roles in animal tissues (6). Because the proportion of AcAc and Ac in circulating ketone bodies is very low and their unstablebilities (6, 49), we will mainly focus on the physiological role of BHB. BHB was considered a passive energy carrier in the early stage (52). But, with the deepening of research in recent years, abundant evidence authenticated its signalling carrier roles, which affect various physiological processes by multiple mechanisms.

### Alternative Energy Source

The glucose supply in animals does not meet the body’s energy requirement in many physiological states, such as neonatal period, fasting, prolonged exercise, pregnancy and lactation, and adherence to low-carbohydrate diets (53, 54). Under these conditions, the stored energy in muscle and liver glycogen and the fatty acids residing in adipose tissues will be mobilized. As the major form of energy storage, adipose tissues, which contain more than 80% stored energy of the body, are catabolized into FFAs through lipolysis and parts of these FFAs are transported to the liver for ketogenesis (7). The hepatic BHB is then distributed *via* blood circulation to metabolically active tissues, including muscle, brain, and heart, then metabolized into Ac-CoA, and eventually ATP in the TCA circle (34) (**Figure 1**). The liver can produce up to 300 g of ketone bodies per day in human bodies, which provides 5~20% of the total energy expenditure (34, 55). In addition, BHB has a higher H: C ratio than pyruvate (2 and 1.3, respectively) and higher reducibility, which means that it yields more free energy per mole of oxygen to fuel ATP production (56) and consequently was thought to produce fewer byproducts of reactive oxygen species (ROS) than glucose or FFAs (57). However, ROS was also thought to be more than a byproduct of mitochondrial terminal oxidation and play an important role in sustaining the function of T cells (58, 59), which might explain why pyruvate instead of BHB was chosen as the main substrate for mitochondrial terminal oxidation in normal state. In short, this alternative energy storage is considered a mechanism developed by animals to adapt to food availability and nutrient stress (34).

### Direct and Indirect Regulators of Various Physiological Processes

Apart from passive energy carriers, BHB is also involved in multiple signalling functions at the cell surface and intracellular, affecting gene expression, lipid and protein metabolism, neuronal function, and metabolic rate by direct or indirect mechanisms (**Table 1**).

**Table 1:** The roles of BHB as signal carriers.

type	mechanism	physiological processes	references
direct	HDACs inhibition	gene expression	(60–62)
	BHB-ylation	gene expression	(63–65)
	FFAR inhibition	sympathetic system	(66, 67)
	HCAR2 activation	lipid metabolism	(68–70)
	plasma member K^+^ channel expansion	NLRP3-induced inflammation	(71, 72)
	VGLUT inhibition	epilepsy	(73, 74)
indirect	feedback inhibition of Ac-CoA	glucose metabolism	(52, 75)
	inhibition of protein succinylation	lipid metabolism	(76, 77)
	NAD^+^ sparing	sirtuin activity	(78–80)
	GABA synthesis	epilepsy	(81, 82)

HDACs, histone deacetylases; BHB-ylation, β-hydroxybutyrylation; FFAR3, free fatty acid receptor 3; HCAR2, hydroxycarboxylic acid receptor 2; NLRP3, NOD-like receptor protein 3; VGLUT, vesicular glutamate transporter; Ac-CoA, acetyl-CoA; NAD^+^, nicotinamide adenine dinucleotide; GABA, gamma-aminobutyric acid.

**Figure 2:**
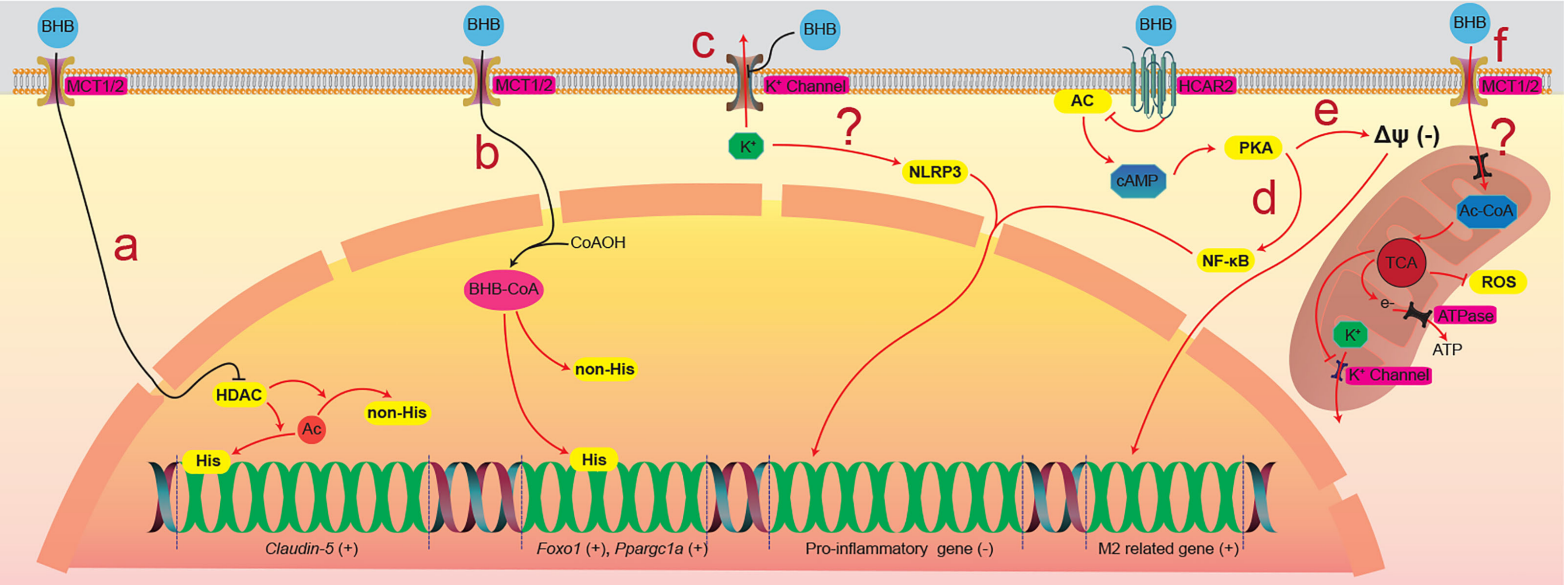
The physiological processes that BHB direct regulates. **(A)** BHB across the plasma membrane enters the nucleus and inhibits HDACs, which participate in the deacetylation of histone and nonhistone proteins, resulting in the increased expression of the *claudin-5* gene in the cardiovascular endothelial cells. **(B)** BHB that enters the nucleus was activated into acetylated BHB and then β-hydroxybutyrates histone and nonhistone proteins, resulting in the increased expression of *Foxo1* and *Ppargc1a* gene in the CD8^+^ T cells. **(C)** BHB holds back the K^+^ channel in the plasma membrane, which maintains the cytoplasmic K^+^ concentration and inhibits the activation of the NLRP3 inflammasome. BHB activates HCAR2, a seven-transmembrane G-protein coupled receptor of the Gi family, and inhibits the activity of the AC/cAMP/PKA signaling pathway. **(D)** the inhibition of the AC/cAMP/PKA signaling pathway decreases the mitochondrial membrane potential and promotes the transcription of M2-related genes in intrahepatic macrophages. **(E)** the inhibition of the AC/cAMP/PKA signaling pathway also inhibits the activation of NF-κB, consequently inhibiting the expression of pro-inflammatory genes in primary rat microglial cells. **(F)** the oxidation of BHB in the mitochondrion closes the channel proteins in the mitochondrial intima, which inhibits the outflow of ROS. BHB, β-hydroxybutyrate; BHBtion, β-hydroxybutyration; MCT, monocarboxylate transporter; HCAR2, hydroxycarboxylic acid receptor 2; HDACs, histone deacetylases; Ac, acetyl group; His/non-His, histone/nonhistone proteins; AC, adenylate cyclase; cAMP, cyclic adenosine monophosphate; PKA, protein kinase A; NF-κB, nuclear factor kappa-B; Ac-CoA, acetyl coenzyme A; TCA, tricarboxylic acid cycle; ROS, reactive oxygen species; ATP, adenosine triphosphate; ATPase, ATP synthase.

BHB was found as a competitive inhibiting catalytic site, directly inhibiting class I histone deacetylases (HDACs) (83), which were thought to participate in the regulation of gene expression by deacetylating lysine residues on histone and nonhistone proteins, such as NF-κB, TP53, MYC, and MYOD1 et al., and consequently regulates corresponding gene expression (84, 85). Li and colleagues found that BHB upregulated *claudin-5* gene expression and ameliorated the diabetes-associated cardiac endothelial hyperpermeability by inhibiting HDAC3 (60) (**Figure 2A**). By lysine β-hydroxybutyrylation, BHB can also directly modify proteins in multiple model organisms, including yeast, fly, rat, and human cells, and regulates gene expression (63). Zhang and colleagues found that the β-hydroxybutyrylation of Lys 9 of histone H3 (H3K9) upregulated the expression of *foxo1* and *ppargc1a*, contributing to the development of CD8^+^ T_mem_ cells (64) (**Figure 2B**). BHB is also the ligand of two cell surface G-protein-coupled receptors, hydroxycarboxylic acid receptor 2 (HCAR2), also called GPR109A (86), and free fatty acid receptor (FFAR), which both were thought to play important roles in metabolism and metabolic diseases (66, 87, 88). Chen and colleagues found that BHB activated the HCAR2 signalling pathway, which increased M2-related gene transcription in intrahepatic macrophages and attenuated liver damage induced by alcohol hepatitis by lowering mitochondrial membrane potential (68) (**Figure 2C**). Liu and colleagues found that BHB activated HCAR2 and inhibited the activation of the NF-κB signaling pathway, which decreased the release of pro-inflammatory cytokines in primary rat microglial cells (15, 16) (**Figure 2D**). There is also evidence indicating that BHB affects the K^+^ channel and modulates potassium flux across the plasma membrane (71, 72), which probably explains why BHB inhibits the activation of NOD-like receptor protein 3 (NLRP3) inflammasome and ameliorates NLRP3 inflammasome-mediated inflammatory diseases (89) (**Figure 2E**). Interestingly, oxidation of BHB was found to close the mitochondrial permeability transition pore, which maintains the electrochemical potential gradient required for ATP generation of oxidative phosphorylation (56). This closure was thought to protect neurons against ROS-induced apoptosis (90, 91) (**Figure 2F**). Besides, by directly inhibiting the neuronal vesicular glutamate transporter (VGLUT), BHB reduces excitatory glutamate neurotransmission without affecting inhibitory gamma-aminobutyric acid (GABA) neurotransmission (92), which is believed to be involved in the process of lowering the seizure of epilepsy by utilizing a ketogenic diet (73). However, some nonnegligible evidence shows that BHB might increase the release of inflammatory factors. For instance, Neudorf et al. found that elevated circulating BHB by oral ketone supplementation significantly increased the levels of IL-1β and IL-6 but not TNF-α or IL-8, through a caspase-1 dependent manner in human monocytes (93). Li et al. also found that, by activating the NF-κB signal pathway, BHB increased the release of pro-inflammatory factors (94). These contradictory results indicate that elevated BHB might have anti-inflammatory or pro-inflammatory effects for different target cells or in a different trigger method (exogenous or endogenous). The mechanisms of these contradictory effects should be the most priority in studying BHB on cell physiology, which is urgent for researchers to solve as soon as possible.

In addition to the above direct modulator roles, BHB also exerts indirect signalling effects by its metabolic intermediates, including but not limited to Ac-CoA, succinyl-CoA, and NAD^+^/NADH, during its catabolism to ATP (95). The catabolism of BHB increases the level of intracellular Ac-CoA (**Figure 1**), which was thought to post-transcriptionally modulate gene expression *via* both enzymatic and nonenzymatic protein acetylation (96). As the alternative energy source, by increasing cytoplasmic citrate and inhibiting the activities of phosphofructokinase (PFK) and pyruvate dehydrogenase (PDH), BHB inhibits glycolysis, the cytoplasmic steps of glucose utilization in many tissues (**Figure 1**), such as the heart, brain, skeletal muscle, and tumours (6, 55, 71, 75), maintaining blood glucose at a necessary level. Besides, Ac-CoA-derived acetylation has extremely extensive influences on multiple cellular compartments, particularly mitochondria and nucleus, which are very sensitive to acetyl-CoA concentration and whose gene expression is readily affected (85). The catabolism of BHB also consumes succinyl-CoA to donate the CoA to AcAc (97) (**Figure 1**). Like acetylation, lysine succinylation also plays an important role in the mitochondria of diverse organisms (76, 98). Its consumption affects the balance of lysine succinylation, which affects the expression of HMGCS2 and other genes (76, 99). NADH utilization during BHB metabolism is different from glucose metabolism. Considering that the cytoplasmic and mitochondrial NAD^+^: NADH equilibrium is thought to be crucial in metabolic disease and ageing (78), this alteration might also explain the anti-ageing effects of the ketone diet. Moreover, BHB also serves as a substrate for synthesizing glutamine and other amino acids in astrocytes (100), which further alters the biosynthesis of the inhibitory neurotransmitter GABA (101). On the whole, the signal molecule actions of BHB involve multiple molecules and pathways, which need to be further deeply investigated.

Referencing the term ‘RNA World’ proposed by Ádám et al. (102), we propose an analogical concept: ‘BHB World’. BHB is a more effective fuel molecular with fewer byproducts and a more regulative intermediate metabolite that regulates multiple metabolisms processes (52) (**Figures 1**, **2** and **Table 1**). In adipose tissues, which are crucial for animals’ survival, FFAs, the precursor of BHB, can be steadily and massively stored and quickly utilized. BHB also connects and regulates multiple physiological processes like a bridge (95). These functions are similar to the performance of RNA as genetic information storage and functional enzymes. Considering these seemingly coincidental similarities, we can speculate that BHB might also act as the main circulation fuel molecular rather than glucose in the early stage of the life evolution process. However, this theory has never been proposed before and needs to be discussed more.

## Barrier Immunoregulatory Role of BHB

The energetic and signalling functions of BHB suggest its extensive physiological activities, such as an immunoregulator. Indeed, accumulating evidence corroborates the important role of BHB on immunoregulation by different mechanisms, including nutrition competition, metabolic reprogramming, immune microenvironment modification, and gene expression regulation, in various tissues, such as peripheral blood, brain, respiratory tract, and digestion tract, which have been well-summarized before (1, 3, 21, 103). However, there are mounts of reports regarding the effects of BHB on the function of the immunological barrier, which has never been reported before in literature. Hence, in this section of the review, we will outline and discuss the regulatory role of BHB on the immunological barrier, mainly mucosa.

**Figure 3:**
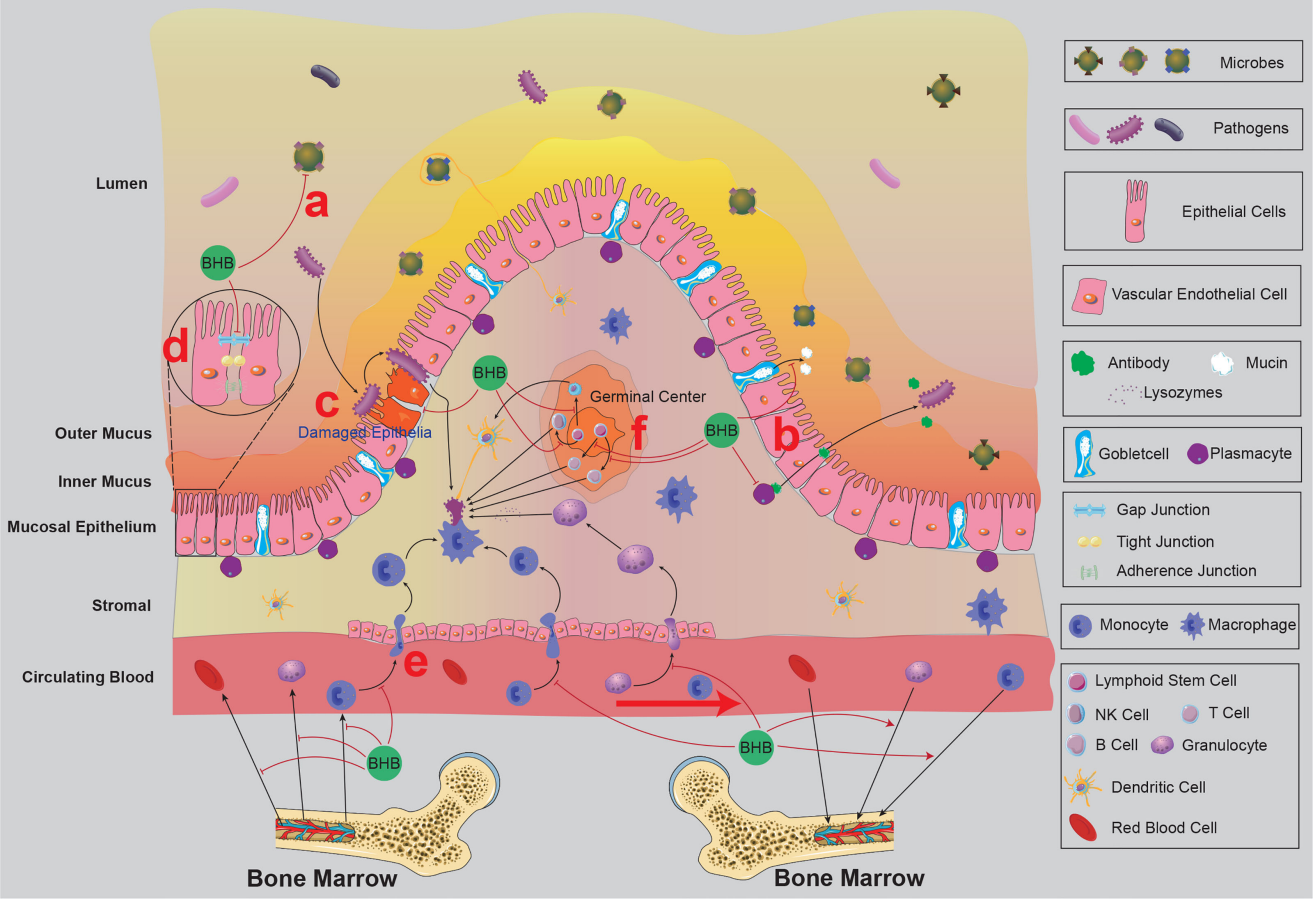
The mucosal immunity of the intestine and the possible effects of BHB on intestinal mucosal immunity. BRC and circulating immune cells from bone marrow circulate in the blood and lymphatic system and then return to the bone marrow. BHB was found to accelerate the homing process of circulating naïve B cells, decrease the expression of CXCL13 (a chemokine that recruits naïve B cells) in the intestinal vascular endothelial cells and stromal cells, and increase the expression of adhesion proteins between vascular endothelial cells, indicating BHB might reduce the chance of circulating immune cells infiltrating into the intestinal mucosa. The germinal centre in the intestine tissue contains multiple kinds of follicular lymphocytes, which are released into the stromal participating in the immune defence process. BHB was found to inhibit the proliferation and differentiation of primary follicular lymphocytes, indicating BHB might reduce the number of follicular lymphocytes in the stromal. There are massive microbes on the surface of the small intestinal mucosa, which prevents the colonization of pathogens (Microbiota Barrier). BHB was found to inhibit the growth of *Bifidobacterium* and *Lactobacillus* in the intestine and alter the microflora community of the nasopharynx, indicating BHB affects the microbiota barrier of mucosal. Covering the surface of mucosal epithelium, a layer of mucus consists of kinds of chemical components (Chemical Barrier), including mucins secreted by goblet cells, antibacterial peptides secreted by epithelial types of cells, and sIgA secreted by plasma cells, et al. BHB was found to affect the thickness and viscosity of the mucosal layer, indicating BHB might affect the secretory function of these secretory cells. The mucosal epithelial cells are woven into a dense network by tight junctions and intermediate junctions, including occlaudin, claudin, E-cadherin, Ep-CAM, et al. This network prevents pathogens from invading the mucosal tissue and resulting in infection (Physical Barrier). BHB affects the expression of adhesion proteins between vascular endothelial cells, indicating that it might also affect the physical barrier of mucosal epithelium. Macrophages, DCs and other immune cells play important roles in recognizing and eliminating the invaded pathogens. BHB was found to decrease the pro-inflammatory activities of granulocytes and macrophages, indicating that BHB might also affect the activities of immune cells in the infected mucosal tissues. BRC, blood-red cell; BHB, β-hydroxybutyrate; sIgA, secretory immunoglobulin; E-cadherin A, epithelial cadherin; Ep-CAM, epithelial cell adhesion molecule; DCs, dendritic cells.

Over 400 m^2^ of mucosa and skin in human bodies are constantly exposed to the environment, harbouring massive microorganisms since birth, and over 70% of immune cells resident in the mucosal system (104). These barriers maintain the equilibrium between the body and the external environment by constructing microbial, chemical, and physical protections (105) (**Figure 3**). Indubitably, these barrier immune systems are the first defence line against infections.

### BHB Affects Microbiota Barrier

The mucosa and skin are colonized by massive commensal, opportunistic pathogenic, and pathogenic microorganisms, covering the epithelial cells and forming a layer of microbiota barrier (106). The commensal microflora competes for ecological niches and nutritional resources with pathogenic microflora, which is thought to inhibit the invasion and proliferation of the latter, indirectly protecting the tissues from infection (107). Importantly, the competition among these microorganisms is homeostatic, which was recently confirmed to be reproducibly influenced by BHB (**Figure 3**).

The mucosal microbiota of the gut is considered an important regulator of the immune system (108, 109). Paoli and colleagues systematically reviewed the relationship between ketogenic diets (KD) and the gut microbiome, showing common consequences: decreasing the α diversity and richness of the gut microflora community and decreasing some specific bacteria (110), indicating the gut microflora regulatory role of BHB. Newell and colleagues found that KD significantly decreased the gut microbiota α diversity of the B6 murine model of autism spectrum disorder, characteristically reducing the relative abundance of *Bifidobacterium* and *Lactobacillus* genera (111). Then Ang and colleagues observed very similar gut microbiome alterations in both human and B6 mice (35). To explore the underlying mechanism, they also conducted both *in vivo* and *in vitro* experiments, showing that BHB directly inhibited the proliferation of the *Bifidobacterium* and most gut-resident anaerobes (35). This direct bacteriostasis of BHB might explain the KD-induced gut microbiota alteration, at least partly. Considering that *Bifidobacterium* is proven to induce the proliferation of pro-inflammatory T_h_17 cells in the intestinal of mice and humans (35, 112), it’s believed that BHB might regulate the gut microflora through some indirect mechanisms, which needs to be verified in further investigations. Even though there seems to be a consensus that BHB has an important influence on the gut microbiome, very limited research directly studies its effects on the gut microflora, and its mechanism is not well characterized either.

The mucosal microbiota in the respiratory tract is also considered an important regulator of the immune system (107, 109). In previous work, by dietary restriction and intravenous injection of exogenous BHB, it was found that elevated circulating BHB significantly increased the α diversity and the richness of the yak nasopharyngeal mucosal microbiota community, contrary to the gut, with an unexpected increase of many bovine respiratory disease-related bacteria, indicating the increased risk of bovine respiratory diseases (13). The prominently changed genus in the experiment was also obviously distinct from those in the gut, suggesting that BHB has a different influence on the digestive and respiratory tract microflora community. This work also implied that hyperketonemia rather than dietary changes were more responsible for the nasopharyngeal mucosal microbiome alteration (13). It must be highlighted that yak is not a canonical model organism, and whether a similar BHB-induced nasopharynx-colonized microorganisms alteration will occur in mice and humans must be verified by further experiments. Based on these results, it can be analogously assumed that BHB also affected the microbiota of the lower respiratory tract. However, apart from this report, few references about the effect of BHB or KD on the respiratory tract can be found.

There are a few essentials to be noticed. Firstly, the short-chain fatty acids (SCFAs) represented by butyrate possess a similar bacteriostatic effect to BHB (108, 113), and they are also mucosal immunoregulatory molecules (113). Ang et al. observed no significant alteration in the level of SCFAs, including butyrate, in their experiment, indicating SCFAs were not involved in the gut microbiota growth inhibition of KD even though butyrate inhibits the growth of *Bifidobacterium* (35). However, more evidence is needed to distinguish the immunoregulatory effects of SCFAs and BHB. The second point is the extremely complex interreactions among these barrier-colonized microorganisms, and this equilibrium will be broken even though a slight alteration in a single kind of microorganism (106). This complexity makes it more difficult to identify the specific effects of BHB on the microbial community. Thirdly, the altered microorganism also affects the immune system where it colonizes. For instance, Tagliabue et al. found that three months of KD significantly decreased the abundance of *Desulfovibrio spp* in patients with Glucose Transporter 1 Deficiency Syndrome. *Desulfovibrio spp* participates in regulating mucus layer inflammatory status, indicating BHB could indirectly affect the immune system by exerting influence on the microflora (114). Finally, the microbiota in the gut and respiratory tract mucosa is important for inducing immune tolerance (109). It was noticed that fasting inhibited the induction of oral tolerance (115). However, whether the inhibition of microflora induced by BHB affects the induction of immune tolerance is uncertain and warrants attention. In short, according to the different effects of BHB on the intestinal and the nasopharynx, it’s reasonable to suspect that BHB exerts its barrier microbiota regulatory role in an unknown tissue-specific way *via* different mechanisms which warrant further investigation.

### BHB Affects Chemical Barriers

The chemical components, such as mucins secreted by goblet cells, antibacterial peptides secreted by epithelial types of cells, and secretory immunoglobulin A (sIgA) secreted by plasma cells et al., are released into the mucosal secretions and form a dense viscoelastic barrier, covering the mucosal epithelial cells and resisting the adhesion and invasion of microorganisms (116, 117), like a city moat (**Figure 3**). Referencing previous works (118, 119), we named these barriers chemical barriers instead of mucus barriers because mucus barriers seem to be used to describe the mucosa only (does not include the skin). Prior works suggested that the richness decrease induced by carbohydrate restriction decreased the gut mucosal mucus layer (120, 121). Besides, Nagai and colleagues found that fasting significantly reduced the production of IgG, IgM, and antigen-specific sIgA (115). These works implied that BHB might also affect the generation of mucus. However, Ang and colleagues noticed KD neither reduced the expression of the *muc2* gene nor the thickness of the mucus layer in gut mucosal (35). Intriguing, two randomized controlled trials also investigated the effect of KE oral administration on athletes’ immune response to exercise, focusing on sIgA secretion, indicating that elevated blood ketones slightly increased sIgA level in saliva (122, 123). Given that fasting and KD are usually accompanied by elevated blood BHB, these heterogeneous results implied that BHB might have complicated effects on the mucosal mucus layer. It’s proposed that the decreased consumption of mucins by microbiota, the altered quantities and activities of immune cells, and the modified secretion and transportation abilities of plasma cells and epithelial cells accounted for these complicated effects on the mucus layer (13, 115, 120). However, these conjectures all need to be further investigated.

### BHB Affects Physical Barriers

The tissue barrier function of the mucosa is provided by the tightly interlaced cell-to-cell network of epithelial cells and intraepithelial lymphocytes (124) and the microvilli of epithelial cells (125). There are tight junctions and intermediate junctions among the epithelial cells and intraepithelial lymphocytes, such as occlaudin, claudin, epithelial cadherin (E-cadherin), and epithelial cell adhesion molecule (Ep-CAM), which weave these cells into a dense network, impeding microbiota invading epithelial tissues and wielding innate immunity function (125, 126) (**Figure 3**). Mounts of studies indicated that prolonged aerobic exercise increased intestinal permeability (127–129). Although no evidence indicated that ketone bodies were involved in the exercise-induced augmented intestinal permeability, it cannot be excluded that BHB contributed to the permeability augmentation. Serino and colleagues found that a high-fat diet-induced hyperketonemia increased the permeability of mice’s gut and resulted in a higher concentration of endotoxin in the blood (130). We hypothesize that the increased intestinal permeability allows more nutrient substances to be transported into the vein by a paracellular pathway to replenish the prolonged exercise-induced over energy consumption. In this process, it is inevitable that some antigens (bacteria) also pass through the epithelial barrier, which results in endotoxaemia. However, ketone bodies were also found to upregulate the expression of endothelial connexin 43 (Cx43) gap junctions of bovine vascular endothelium (131), which decreased the permeability. This contradiction could be explained by the different functions of these two types of mucosal. The intestinal epithelium absorbs nutrients from the outsides while the vascular provides tissues with nutrients. These contradictive responses to elevated BHB concentration have the same aim: maintaining a necessary blood glucose concentration. Anyway, these clues indicated that BHB affected the junctions among mucosal tissue cells. Although the direct evidence is lacking, it is an attractive research direction to investigate the different influences of BHB on the permeability between the digestive tract and respiratory tract mucosal epithelium, even between different sections. Furthermore, the mucociliary clearance produced by epithelial microvilli also wields an important role in the resistance against microbiota adhesion and invasion (125). However, there is no study regarding the effects of ketone bodies on epithelial microvilli function, which is worthy of investigating.

Apart from the mucosa, some other tissues also act as barriers, including the skin, BBB, blood-embryo barrier, blood-testis barrier, blood-ocular barrier, and vascular barrier endothelium, et al. Skin, a specific mucosal barrier, was reported to be influenced by KD, especially in the artificially induced murine psoriasis model (132, 133). A high-fat diet significantly reduced the expression levels of BBB transporters and tight-junction proteins (claudin-5, occludin) in mouse brain capillaries (134), which supported our previously mentioned explanation. Another study revealed that elevated BHB concentration also increased the retinal outer nuclear layer Cx43 expressions, which improved retinal permeability and homeostasis (69). The researches regarding the effect of ketone bodies on these specific barriers are quite limited and warrant more attention, especially on the blood-embryo barrier and vascular endothelium.

It’s important to realize that these three levels of barriers discussed above are just a part of the mucosal immune system, which also contains the innate immune cells in the stroma from the circulating blood or the germinal centre (**Figure 3**). Since these innate immune cells are also distributed in other non-mucosal tissues (22, 135), the effects of BHB on these cells were not discussed in this review. Collectively, BHB is an important regulator for the barrier immune system, but these mechanisms are still confusing.

Even though many eyes focused on the immunomodulatory roles of BHB and accumulating literature was published, this knowledge gap is still huge and needs to be deeply investigated. For instance, are there any differences between the effects of endogenous and exogenous BHB? Is it feasible to apply the immunomodulatory effect of BHB to the treatment of respiratory inflammation? Is the role of BHH consistent in the respiratory and digestive tract? All these queries need to be answered by the efforts of a large number of researchers.

## Concluding Remarks

BHB is an endogenous natural small molecule that presents in almost all life realms, connecting glycometabolism, lipometabolism and proteinmetabolism. During its metabolism as energy molecular, NAD^+^ is oxidated to NADH, the succinyl-CoA is consumed, and lots of Ac-CoA is generated, inhibiting glycolysis and affecting the metabolic environment of extrahepatic cells. Together with its protein and gene modification function, BHB can alter the metabolism, gene expression, and post-transcriptional modification of cells, especially the immune cells. Because BHB is a signal of energy deficiency and a more reductive substrate molecule, elevated blood BHB concentration will guide the body to shut unnecessary physiological processes down, including altering the permeabilities of many endothelium and epithelium to support pivotal processes and reducing the activities of some high energy-consuming cells (plasma cells and goblet cells). These alterations consequently affect the mucosal immune systems, showing great potential in treating mucosal and skin-related diseases. However, the detailed mechanisms and progresses behind these alterations are not fully understood and deserve further investigation.

## Author Contributions

JQ, JF and XY wrote this manuscript, and all authors reviewed and corrected this manuscript. We appreciate Dr. Wenqing Zhang and JZ for their assistance in drawing sketch maps.

## Funding

This work was supported by the China Agriculture Research System of MOF and MARA (Beef Cattle/Yak, CARS-37) and the Sichuan beef cattle innovation team of the national modern agricultural industry technology system (SCCXTD-2020-13).

## Conflict of Interest

The authors declare that the research was conducted in the absence of any commercial or financial relationships that could be construed as a potential conflict of interest.

## Publisher’s Note

All claims expressed in this article are solely those of the authors and do not necessarily represent those of their affiliated organizations, or those of the publisher, the editors and the reviewers. Any product that may be evaluated in this article, or claim that may be made by its manufacturer, is not guaranteed or endorsed by the publisher.
